# Disease spectrum and outcomes among elderly patients in two tertiary hospitals in Dar es Salaam, Tanzania

**DOI:** 10.1371/journal.pone.0213131

**Published:** 2019-10-10

**Authors:** Basil Tumaini, Patricia Munseri, Kisali Pallangyo

**Affiliations:** Department of Internal Medicine, Muhimbili University of Health and Allied Sciences, Dar es Salaam, Tanzania; Azienda Ospedaliero Universitaria Careggi, ITALY

## Abstract

**Background:**

There has been an increase in the number of individuals aged ≥60 years in Tanzania and in sub Saharan Africa in general due to improved survival. However, data is scarce on the disease burden and outcomes following admission in this population. We herein describe the pattern of diagnoses, outcomes and factors associated with the outcomes among elderly patients admitted at Muhimbili National Hospital (MNH) and Jakaya Kikwete Cardiac Institute (JKCI) medical wards.

**Methodology:**

From October to December 2017, we consecutively enrolled patients aged ≥60 years (elderly) admitted to the MNH and JKCI medical wards. The ICD 10 was used to code for disease diagnosis at discharge or death. The Modified Barthel index was used to assess for functional activity on admission and at discharge.

**Results:**

We enrolled 336 (30.1%) elderly participants out of 1301 medical admissions. The mean age ± SD was 70.6 ± 8.9 years; 169 (50%) were female and the average number of diagnoses was 2 per participant. The most common diagnoses were: hypertension 151 (44.9%), stroke 106 (31.5%), heart failure 62 (18.5%), pneumonia 60 (17.9%), diabetes mellitus 58 (17.3%) and chronic kidney disease 55 (16.4%). The median duration of hospital stay was 5 (IQR 3–10) days and in-hospital mortality was 86 (25.6%), 56 (65%) deaths were due to non-communicable diseases and 48 (55.8%) deaths occurred within 72 hours of hospitalization. A modified Barthel score ≤20 on admission was associated with an OR 15.43 (95% CI: 7.5–31.7, p<0.001) for death.

**Conclusion:**

Elderly patients constituted a significant proportion of medical admissions at MNH and JKCI with high in-hospital mortality. A modified Barthel index score ≤20 during admission is associated with mortality and can be used to identify patients requiring special attention.

## Introduction

The average life expectancy of a Tanzanian at birth has improved from 49.6 years during the period of 1990–1995 to 66.7 years from 2015, largely due to the success in controlling HIV/AIDS. This, together with declining fertility, is resulting in population aging which is a worldwide phenomenon. Population aging is projected to have profound socioeconomic consequences in the not-too-distant future [1].

The improved survival is coupled with an increase in the proportion of elderly patients admitted due to medical conditions in developing countries [2–5]. The rapid increase in the number of older patients is predicted to overwhelm health care systems as the elderly mostly encounter chronic diseases [6]. An increase in age is not only associated with co-morbidities and disability, management of the elderly may be associated with altered therapeutic responses and adverse reactions [7]. Because of such complexities, management of elderly is best done by a multidisciplinary team with high levels of personnel including geriatricians who are scarce in many developing countries and calls for adequate financial resources [5]. Hence medical management among the elderly poses significant challenges in the developing world. Despite the fact that the elderly is a highly dynamic group, the group still remains understudied in many developing countries and hence evidence for guiding policy interventions is lacking.

The pattern of diseases among geriatric patients admitted to the medical wards in developing countries has been evolving from infectious diseases to predominantly non-communicable diseases [2,4,5,8]. This makes periodic evaluation of the changing patterns imperative to guide practice and appropriate policies. It has also been shown that physical disability and impaired capacity for self-care has increased rapidly among the elderly due to comorbidity [2]. Hence evaluation of the functional status is an important element of any geriatric assessment. Evaluation of functional status using simple yet invaluable activities of daily living (ADL) tools is not part of the standard of care in Tanzania and hence the usability of these tools as well as information on the magnitude of disability is lacking.

This study aimed at determining proportion of individuals aged ≥60 years among medical admissions, their demographics, diseases, hospital outcomes as well as associated factors, at two tertiary hospitals in Dar es Salaam Tanzania. We compare data from this study with data published in 1994 from the same center.

## Materials and methods

### Ethics statement

Ethical approval was obtained from the Muhimbili University of Health and Allied Sciences Institutional Review Board. Muhimbili National Hospital and Jakaya Kikwete Cardiac Institute administration granted permission to conduct the study in the respective institutions. Written informed consent was obtained from all study participants /caretakers for patients who were unable to provide consent prior to enrolment into the study. All participants received treatment as per hospital standard operating guidelines.

### Study design and population

This study was conducted at two public tertiary referral hospitals located in Dar es Salaam, Tanzania: Muhimbili National Hospital (MNH) and Jakaya Kikwete Cardiac Institute (JKCI) from 1^st^ October to 5^th^ December 2017. MNH has a bed capacity of 1500, the medical ward has a bed capacity of 160. Patients admitted in the medical wards include all spectra of medical sub-specialties including: neurology, gastroenterology, infectious disease, pulmonology, rheumatology, dermatology, endocrinology, hematology, and nephrology. JKCI has a bed capacity of 103 and admits patients with cardiac diseases. These hospitals have highly skilled staff and advanced diagnostic facilities. We consecutively enrolled patients aged ≥60 years who were admitted through MNH Emergency Medicine Department (EMD) to the medical wards at MNH and JKCI.

### Data collection

The investigator (B.T.) administered interviewer-based structured questions to the study participant/caretaker for participants who were unable to communicate. We asked questions on socio-demographic characteristics and functional status; performance of ADL was adapted from the modified Barthel Index [9,10]. The Barthel Index questions were administered at admission and discharge. Guidelines for the Barthel Index were employed to ensure what was recorded is what the patient does, using the best available evidence. Unconscious patients were scored 0 throughout. Patients were followed up until discharge from the hospital or death. Final diagnoses for each participant were recorded from the hospital charts and thereafter coded using ICD-10 [11] at the time of outcome. Length of hospital stay was computed as the difference between the date of admission and the date of discharge/death for each participant. The underlying cause of death was obtained from the death certificates. We studied factors such as socio-demographic characteristics, diagnoses and functional status on admission as measured by the modified Barthel ADL index and their association with mortality.

### Statistical methods

Data was entered into EpiData version 3.1 and thereafter exported into IBM^®^ SPSS^®^ Statistics version 23 for analysis. Qualitative variables such as sex, level of education, marital status, hospital outcome, discharge diagnosis, cause of death and health insurance status were summarized as frequency and proportions. Differences in proportions across groups were compared using Chi-square test or Fisher’s exact test. Quantitative variables such as length of hospital stay, age, and modified Barthel ADL score at admission and at discharge were summarized as means and standard deviation. The student’s t-test was used to compare quantitative variables among participants who survived to those who died. A logistic regression analysis was performed to identify independent risk factors for mortality. All factors with p value of <0.2 in bivariate analysis were included in the multivariate analysis model. Statistical significance was accepted at p<0.05.

## Results

**Fig 1:**
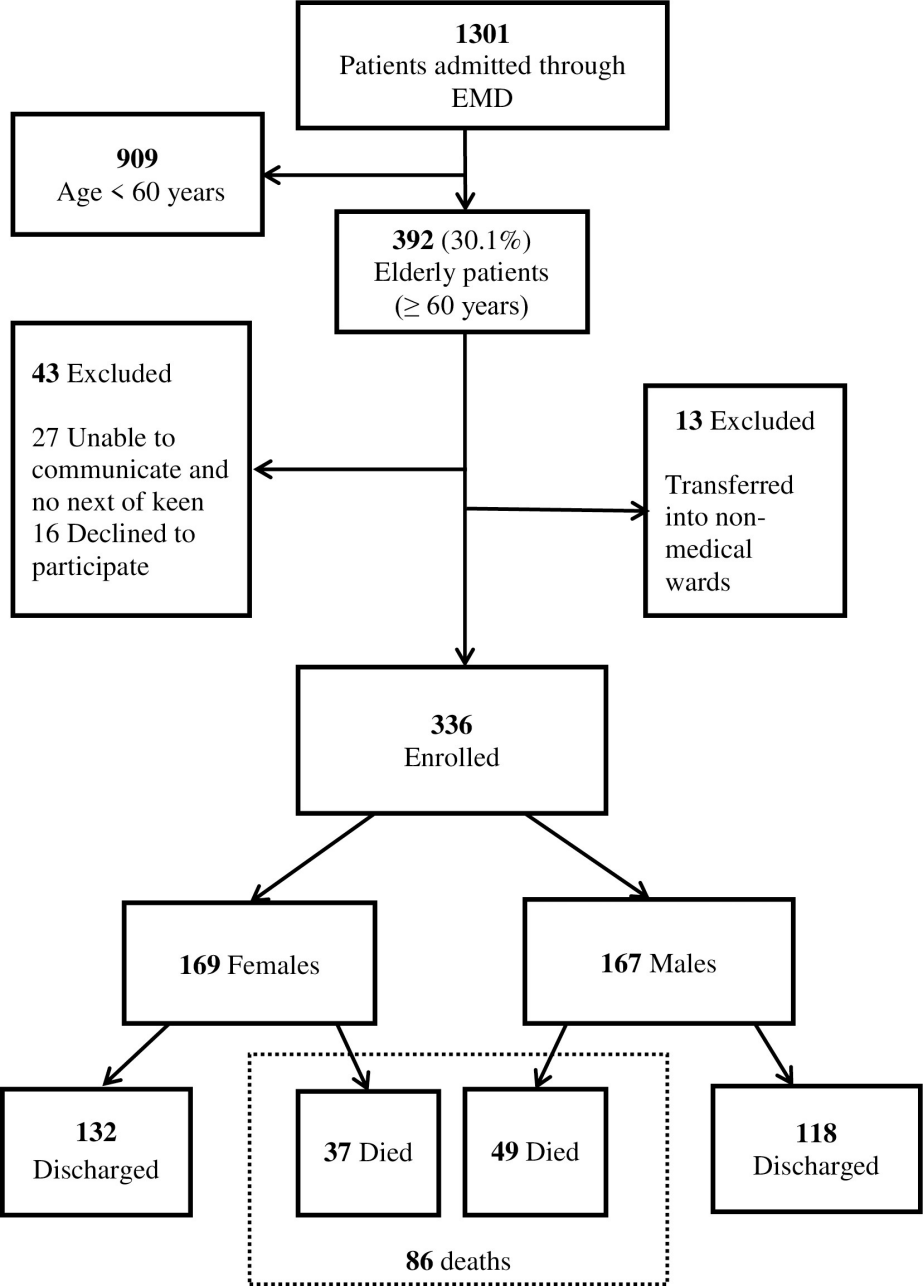
Consort diagram. EMD = Emergency medicine department.

A total of 1301 patients were admitted to the MNH and JKCI medical wards during the three-month study period, out of which 392 (30.1%) were ≥60 years of age. Details of enrolment are shown in Fig 1. In brief, 56 (14%) patients were excluded; 336 participants were enrolled into the study.

### Socio-demographic characteristics of study participants

Table 1 summarizes the socio-demographic characteristics of the study participants. There were 169 (50.3%) females enrolled, the mean age (± SD) for enrolled participants was 70.6 (± 8.9) years, 180 (53.6%) were in the age group 60–69 years and 132 (39.3%) had health insurance. About half of the study participants had at least primary education. However, the proportion of females who had no primary education was 26.6% (45/169) compared to 11.4% (19/167) among males, p<0.001.

**Table 1:** Socio-demographic characteristics of the elderly at admission.

Variable	Total N = 336	Female	Male	*p-value
		n = 169 (50.3%)	n = 167 (49.7%)	
**Age (years**)				
60–69	180 (53.6%)	85 (50.3%)	95 (56.9%)	0.374
70–79	95 (28.3%)	48 (28.4%)	47 (28.1%)	
80–89	51 (15.2%)	29 (17.2%)	22 (13.2%)	
90+	10 (3.0%)	7 (4.1%)	3 (1.8%)	
Mean Age ± SD	70.6 ± 8.9	71.1 ± 9.4	70.1 ± 8.3	0.297
**Health insurance coverage**				
None	204 (60.7%)	108 (63.9%)	96 (57.5%)	
NHIF	130 (38.7%)	60 (35.5%)	70 (41.9%)	0.481
NSSF	2 (0.6%)	1 (0.6%)	1 (0.6%)	
**Level of education**				
No formal education	64 (19.0%)	45 (26.6%)	19 (11.4%)	<0.001
Primary	168 (50.0%)	85 (50.3%)	83 (49.7%)	
Secondary	77 (22.9%)	31 (18.3%)	46 (27.5%)	
College and University	27 (8.0%)	8 (4.7%)	19 (11.4%)	
**Marital status**				
Single	7 (2.1%)	3 (1.8%)	4 (2.4%)	
Married	228 (67.9%)	102 (60.4%)	126 (75.4%)	
Cohabiting	5 (1.5%)	0 (0.0%)	5 (3.0%)	
Divorced	29 (8.6%)	14 (8.3%)	15 (9.0%)	
Widow/Widower	67 (19.9%)	50 (29.6%)	17 (10.2%)	<0.001

* p-value for the difference between male and female sex in various characteristics

SD: standard deviation; NHIF: National Health Insurance Fund; NSSF: National Social Security Fund.

### Diagnosis of the study participants

Table 2 summarizes the diagnosis of the study participants, 73 (21.7%) had a single diagnosis while 263 (78.3%) had more than one diagnosis with an average of 2.3 diagnoses per study participant. The common diagnoses were hypertension 151 (44.9%), stroke 106 (31.5%), heart failure 62 (18.5%), pneumonia 60 (17.9%), diabetes mellitus 58 (17.3%) and chronic kidney disease (CKD) 55 (16.4%). HIV was diagnosed in 16 (4.8%) of the study participants. Frequent comorbid conditions were stroke and hypertension in 73 (21.7%), diabetes mellitus and hypertension in 38 (11.3%), CKD and diabetes mellitus or hypertension in 34 (10.1%), stroke and pneumonia in 32 (9.5%), and heart failure and hypertension in 28 (8.3%) of the study participants. Overall, the proportion of non-communicable diseases was 645/780 (82.7%).

**Table 2:** Diagnoses of the study participants (N = 336).

ICD 10 codes**	Diagnoses	Cases (% of cases)*	Proportion of diagnoses
I10 & I15	Hypertension	151 (44.9%)	19.4%
I63 & I61	Stroke (Ischemic, hemorrhagic)	106 (31.5%)	13.6%
I50.9, I25.5, I42.0	Heart failure	62 (18.5%)	7.9%
J18	Pneumonia	60 (17.9%)	7.7%
E11	Type 2 diabetes mellitus	58 (17.3%)	7.4%
N18	Chronic kidney disease (CKD)	55 (16.4%)	7.1%
C22–95.9	Malignant neoplasms	38 (11.3%)	4.9%
D64.9	Anemia	36 (10.7%)	4.6%
E87	Electrolyte disorders	20 (6.0%)	2.6%
B20	HIV disease	16 (4.8%)	2.1%
K27	Peptic ulcer disease	14 (4.2%)	1.8%
N39.0	Urinary tract infection	11 (3.3%)	1.4%
B54	Malaria	7 (2.1%)	0.9%
A15-19	Tuberculosis	6 (1.8%)	0.8%
B90	Sequelae of tuberculosis	5 (1.5%)	0.6%
Multiple	Other diagnoses**	135 (40.2%)	17.3%
		780 (232.10%)	100%

* Some participants had more than one disease and hence the percentage of cases adds to more than 100%.

** Refer Supporting Information (S1 Table) for ICD-10 codes employed to group the data.

HIV: human immunodeficiency virus.

### Hospital outcomes, duration of hospital stay and time to death

Out of the 336 enrolled study participants, 250 (74.4%) were discharged home; 6 (1.8%) of these against medical advice. The overall in-hospital mortality was 86 (25.6%). The commonest underlying causes of mortality are shown in Table 3. Non-communicable diseases were the cause of death in 56 (65%) patients.

**Table 3:** Underlying cause of death by sex (N = 86).

ICD 10 Codes	Underlying cause of death	Female	Male	Total	p-value
I63, I61	Stroke	9 (24.3%)	16 (32.7%)	25 (29.1%)	0.400
J18	Pneumonia	11 (29.7%)	7 (14.3%)	18 (20.9%)	0.081
I50.9, I42.0, I25.5, I49.9	Heart Failure	10 (27.0%)	8 (16.3%)	18 (20.9%)	0.227
N18	Chronic kidney disease	2 (5.4%)	5 (10.2%)	7 (8.1%)	0.694
A41.9	Sepsis*	1 (2.7%)	4 (8.2%)	5 (5.8%)	0.385
E87	Metabolic disorders	1 (2.7%)	3 (6.1%)	4 (4.7%)	0.631
G03.9	Meningitis	2 (5.4%)	1 (2.0%)	3 (3.5%)	0.575
C85, C95.9	Malignant neoplasms	0 (0.0%)	2 (4.1%)	2 (2.3%)	0.504
B54, A15, K74.60	Other causes	1 (2.7%)	3 (6.1%)	4 (4.7%)	0.631
	**Total deaths**	**37 (21.9%)**	**49 (29.3%)**	**86 (25.6%)**	**0.118**

* Excluding pneumonia and meningitis

The median duration of hospital stay for the survivors was 5 (IQR: 3–10) days, 162 (64.8%) participants were discharged within 7 days from admission and 222 (88.8%) were discharged within 14 days. Four study participants (1.6%) had a hospital stay of more than 4 weeks. The median time from admission to death was 3 (IQR: 1–8) days, 48 (55.8%) deaths occurred within three days of hospitalization.

**Fig 2:**
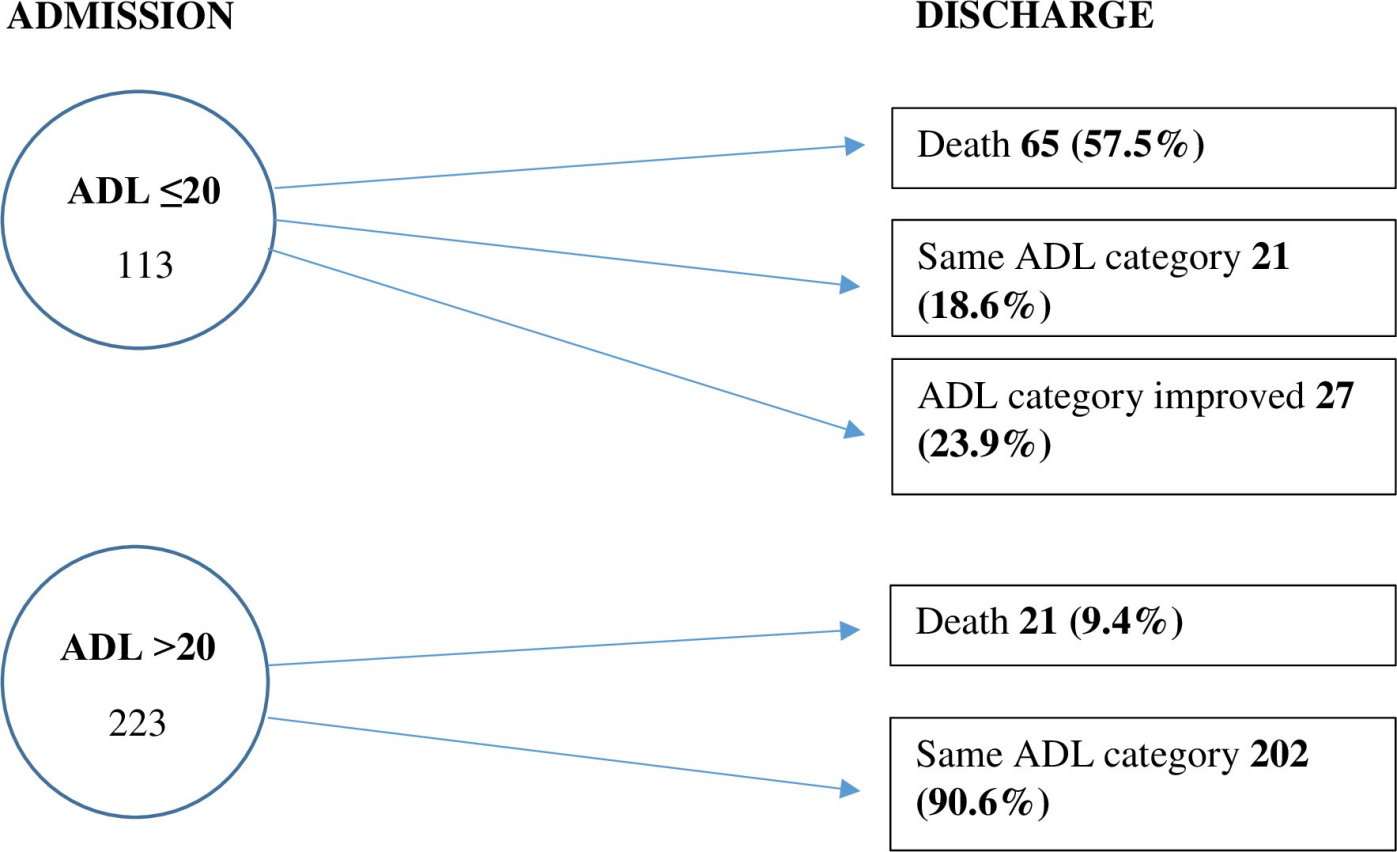
Modified Barthel index scores at admission and discharge.

Overall, 113 (33.6%) participants had modified Barthel index (MBI) scores of ≤20 (total dependence on performance of activities of daily living [ADL]) on admission. Of these 113 patients, 65 (57.5%) died in hospital, 21 (18.6%) still had MBI scores of ≤20 and 27 (23.9%) patients had MBI scores of ˃20 at discharge from hospital as shown in Fig 2. The median MBI score on admission among those who died was 12 (total dependence), IQR: 0–20.5, and was 63 (moderate dependence), IQR: 28–84, among survivors, p< 0.001.

### Factors associated with the hospital outcomes

The mean age of study participants who died in hospital was 72.7 (±9.1) years compared to 69.9 (±8.7) years for those who were discharged home, p = 0.013. The mean MBI score on admission was higher in participants who survived compared to those who died, 57.4 vs 16.9 respectively, p< 0.001. Factors independently associated with mortality were total dependency on admission (MBI score ≤20), adjusted OR 15.43, 95% CI: 7.52–31.66 (p<0.001) and male sex, adjusted OR 1.89, 95% CI: 1.01–3.53, p = 0.046 (Table 4).

**Table 4:** Factors associated with mortality among the 336 study participants.

Variable	Total N = 336	Mortality n = 86	OR (95% CI)	p-value	Adjusted OR (95% CI)	p-value
Modified Barthel Index*						
≤20	113	65 (57.5%)	13.03 (7.26–23.36)	<0.001	15.43 (7.52–31.66)	<0.001
>20	223	21 (9.4%)	1		1	
**Sex**						
Male	167	49 (29.3%)	1.48 (0.90–2.43)	0.119	1.89 (1.01–3.53)	0.046
Female	169	37 (21.9%)	1		1	
**Age [continuous]**			1.03 (1.01–1.06)	0.014	1.04 (1.00–1.08)	0.054
**Has health insurance**						
No	204	60 (29.4%)	1.70 (1.01–2.87)	0.048	1.29 (0.68–2.44)	0.444
Yes	132	26 (19.7%)	1		1	
**Marital status**						
Single	7	1 (14.3%)	1			
Married/Cohabiting	233	59 (25.3%)	2.03 (0.25–17.66)	0.515		
Previously married	96	26 (27.1%)	2.23 (0.26–19.41)	0.468		
**Level of education**						
No formal education	64	17 (26.6%)	1.99 (0.92–4.29)	0.080	1.06 (0.37–3.06)	0.909
Primary education	168	53 (31.5%)	2.54 (1.36–4.73)	0.003	1.70 (0.81–3.59)	0.163
Secondary +	104	16 (15.4%)	1		1	
**Stroke**						
No	225	45 (20.0%)	1		1	
Yes	111	41 (36.9%)	2.34 (1.41–3.88)	0.001	0.63 (0.31–1.31)	0.216
**Pneumonia**						
No	276	63 (22.8%)	1		1	
Yes	60	23 (38.3%)	2.10 (1.16–3.80)	0.014	1.19 (0.57–2.49)	0.649
**Heart failure**						
No	273	66 (24.2%)	1			
Yes	63	20 (31.7%)	1.46 (0.80–2.65)	0.216		
**Chronic kidney disease**						
No	282	72 (25.5%)	1			
Yes	54	14 (25.9%)	1.02 (0.53–1.99)	0.952		
**Hypertension**						
No	183	49 (26.8%)	1			
Yes	153	37 (24.2%)	0.87 (0.53–1.43)	0.588		
**Diabetes mellitus**						
No	278	75 (27.0%)	1			
Yes	58	11 (19.0%)	0.63 (0.31–1.29)	0.206		
**Malaria**						
No	329	84 (25.5%)	1			
Yes	7	2 (28.6%)	1.17 (0.22–6.13)	0.855		
**Malignant neoplasms**						
No	300	79 (26.3%)	1			
Yes	36	7 (19.4%)	0.68 (0.28–1.60)	0.373		
**HIV**						
No	321	80 (24.9%)	1		1	
Yes	15	6 (40.0%)	2.01 (0.69–5.82)	0.199	3.33 (0.84–13.20)	0.088

* Modified Barthel index at admission

HIV: human immunodeficiency virus disease; OR: odds ratio.

## Discussion

Elderly patients comprised a third of all admissions at the JKCI and MNH medical wards despite the fact that they comprise 8.3% of the individuals aged 15 years and above in the Tanzanian general population [12]. This figure is much higher than what was observed in 1998 at MNH whereby individuals aged 55 years and above constituted 14.3% of hospitalizations [13]. The increased proportion of medical admissions among the elderly at MNH/JKCI may be due to an increase in the number of elderly in the general population over the last three decades that has been coupled by an increase in life expectancy [1]. Secondly, elderly people face an increased burden of non-communicable diseases (NCDs). Between 1990 and 2010, NCDs increased by 46 percent in sub-Saharan Africa [14].

The mean age of the study participants was similar to other recent studies among elderly patients in Africa [5]. In this study, there was a balance between elderly males and females admitted contrary to previous studies whereby elderly males formed a greater proportion of hospital medical admissions [2–4,13]. All this has been occurring despite the fact that females tend to have a higher life expectancy compared to males and the fact that the proportion of females is larger compared to males [15,16]. Findings from our study might reflect a change in social dynamics such as improved gender equity in access to health care. We have shown that literacy rate among the elderly was higher than that reported by previous studies. Studies done two to three decades ago had shown that the majority of elderly patients had no formal education[2,3], in this study 81% had attained at least primary education which may be the result of education policies that were implemented by the Tanzanian government to ensure primary education for all.

This study shows that more than three-quarters of the diagnoses were non-communicable diseases. Similar findings have been reported from other countries in sub-Saharan Africa [3,5,17]. In this study, the most frequent single diagnoses were hypertension, stroke and heart failure. These findings were expected given that aging is associated with increasing arterial stiffness and vascular resistance which leads to increasing blood pressure, strokes and myocardial ischemia, declining glomerular filtration rate and impaired response to antidiuretic hormone [18–20]. In addition, the presence of one of these diseases may be a risk factor for another, for example, hypertension is a risk factor for heart failure; diabetes mellitus and hypertension form a significant risk for chronic kidney disease and stroke [21,22]. Indeed similar pattern of diseases among elderly medical admissions has been reported from other centers [3–5].

Infectious diseases like malaria formed a smaller proportion of admissions. The same trend was observed in previous studies [2,3,5] and may be due to the fact that malaria incidence is declining [23,24] and/or because patients with uncomplicated malaria would normally not be referred to a tertiary hospital. HIV diagnosis rate observed is consistent with the current HIV prevalence of 4.7% in Dar es Salaam adult population [25]. Lower HIV diagnosis rates ranging from 0–1.1% were reported in studies involving teaching hospitals in Nigeria, Sudan and northern Tanzania [5].

The median duration of hospitalization was similar to that reported from another tertiary center in Tanzania [5]. However, compared to studies conducted in other centers in Africa and elsewhere [4,5], the duration found in our study was shorter. Given that 8.4% of the study participants were discharged home with total dependency in ADL and the scarcity of home-based care services for diseases other than HIV/AIDS, elderly patients who are discharged home with such severe disability may be at an increased risk of unfavorable outcomes including death. Since the duration of hospital stay is often a trade-off between the standard of care and socioeconomic burden on the patient and the health care system, a study is needed to evaluate and formulate optimum criteria for discharge.

We found that more than a half of deaths among elderly patients admitted to the medical wards occurred during the first 3 days of hospitalization. High initial mortality has also been reported in another study from Nigeria [4]. The high mortality, especially in the first few days of hospitalization, could be due to delays in seeking medical care and/or severity of the presenting clinical condition. Indeed, the in-hospital mortality was significantly lower among patients with health insurance compared to those without. Participants who had health insurance may be at an advantage in that they are able to access better and early hospital care and might also have a better socioeconomic status.

The observed in-hospital mortality of 25.6% is similar to that reported from other centers in Tanzania and other African countries [5,26]. However, the mortality was four times higher compared to in-hospital mortality in the United Kingdom [5]. The observed difference may be due to economic and other barriers to accessing health care leading to delays in diagnosis and treatment. In this study, over 60% of the study participants had no health insurance and were required to pay out of pocket before they could access health care. This creates a major barrier to economically disadvantaged patients. The high early mortality also signals the need for reviewing acute management of medical conditions in elderly patients.

The most important factor associated with mortality was total dependency on activities of daily living at admission similar to the findings from other studies [27–29].

### Strength of the study

This study had an adequate sample size to provide current data on disease burden, diagnosis, and factors associated with the disease outcome in elderly patients admitted in the two hospitals. The prospective recruitment of the study participants ensured completeness of data obtained. Use of ICD-10 allowed for standardized coding and reporting of diseases. The Modified Barthel ADL index, recommended for use in other settings, was used to evaluate functional status at admission and at discharge.

### Study limitations

Data from this study including assessment of functional status using ADL scores was done in experimental/ non-routine conditions, in tertiary hospital facilities; feasibility of performing such assessment in routine conditions may be different.

The findings of this study are from tertiary level care facilities and may not be applicable to lower level care facilities. The study was not designed to follow-up individuals outside the hospital and hence doesn't provide outcomes beyond the date of hospital discharge to determine, for example, if the discharges are premature or not.

## Conclusions

We have shown that elderly patients constitute a significant proportion of the medical admissions at MNH and JKCI and that mortality was high especially during the first week of hospitalization. Non-communicable diseases accounted for over three-quarters of the diagnoses among elderly medical patients. Modified Barthel index score ≤20 at admission was an independent risk factor for a poor in-hospital outcome and can, therefore, be used to identify patients who require special attention.

## Supporting information

S1 Table ICD-10 diagnosis categories: frequencies. (DOCX)
