# Supplementary material for: Disease spectrum and outcomes among elderly patients in two tertiary hospitals in Dar es Salaam, Tanzania
Source: PLoS One. 2019 Oct 10;14(10):e0213131. doi: 10.1371/journal.pone.0213131 (PMC6786655; doi:10.1371/journal.pone.0213131)
Supplement: S1 Table — (DOCX) [file pone.0213131.s001.docx]

**S1 Table. ICD-10 diagnosis categories: frequencies**

|  | Responses | | Percent of Cases |
| --- | --- | --- | --- |
|  | N | Percent |  |
| I10 Essential (primary) hypertension | 149 | 19.1% | 44.3% |
| I15.9 Secondary hypertension, unspecified | 2 | .3% | .6% |
| I63 Cerebral infarction | 91 | 11.7% | 27.1% |
| I61 Nontraumatic intracerebral hemorrhage | 15 | 1.9% | 4.5% |
| I50.9 Heart failure, unspecified | 32 | 4.1% | 9.5% |
| I25.5 Ischemic cardiomyopathy | 17 | 2.2% | 5.1% |
| I42.0 Dilated cardiomyopathy | 13 | 1.7% | 3.9% |
| J18 Pneumonia, unspecified organism | 60 | 7.7% | 17.9% |
| E11 Type 2 diabetes mellitus | 56 | 7.2% | 16.7% |
| E11.0 Type 2 diabetes mellitus with hyperosmolarity | 2 | .3% | .6% |
| N18 Chronic kidney disease (CKD) | 55 | 7.1% | 16.4% |
| C95.9 Leukemia, unspecified | 9 | 1.2% | 2.7% |
| C34 Malignant neoplasm of bronchus and lung | 7 | .9% | 2.1% |
| C22 Malignant neoplasm of liver and intrahepatic bile ducts | 6 | .8% | 1.8% |
| C85 Other specified and unspecifiedtypes of non-Hodgkin lymphoma | 4 | .5% | 1.2% |
| C61 Malignant neoplasm of prostate | 3 | .4% | .9% |
| C90.0 Multiple myeloma | 3 | .4% | .9% |
| C53.9 Malignant neoplasm of cervix uteri, unspecified | 2 | .3% | .6% |
| C18 Malignant neoplasm of colon | 1 | .1% | .3% |
| C24.9 Malignant neoplasm of biliary tract, unspecified | 1 | .1% | .3% |
| C46 Kaposi's sarcoma | 1 | .1% | .3% |
| C71 Malignant neoplasm of brain | 1 | .1% | .3% |
| D64.9 Anemia, unspecified | 36 | 4.6% | 10.7% |
| E87 Other disorders of fluid, electrolyte and acid-base balance | 20 | 2.6% | 6.0% |
| B20 Human immunodeficiency virus [HIV] disease | 16 | 2.1% | 4.8% |
| K27 Peptic ulcer, site unspecified | 14 | 1.8% | 4.2% |
| N39.0 Urinary tract infection, site not specified | 11 | 1.4% | 3.3% |
| B54 Unspecified malaria | 7 | .9% | 2.1% |
| A15 Respiratory tuberculosis | 3 | .4% | .9% |
| A18.89 Tuberculosis of other sites | 3 | .4% | .9% |
| B90 Sequelae of tuberculosis | 5 | .6% | 1.5% |
| N40 Benign prostatic hyperplasia | 12 | 1.5% | 3.6% |
| A41.9 Sespis, unspecified organism | 10 | 1.3% | 3.0% |
| I11 Hypertensive heart disease | 10 | 1.3% | 3.0% |
| I49.9 Cardiac arrhythmia, unspecified | 8 | 1.0% | 2.4% |
| I20 Angina pectoris | 7 | .9% | 2.1% |
| J45 Asthma | 7 | .9% | 2.1% |
| K92.2 Gastrointestinal hemorrhage, unspecified | 6 | .8% | 1.8% |
| E13.621 Other specified diabetes mellitus with foot ulcer | 6 | .8% | 1.8% |
| N17 Acute kidney failure | 5 | .6% | 1.5% |
| A09 Infectious gastroenteritisand colitis, unspecified | 4 | .5% | 1.2% |
| G03.9 Meningitis, unspecified | 4 | .5% | 1.2% |
| I21 Acute myocardial infarction | 4 | .5% | 1.2% |
| I08 Multiple valve diseases | 4 | .5% | 1.2% |
| G45 Transient cerebral ischemic attacks and related syndromes | 4 | .5% | 1.2% |
| G20 Parkinson's disease | 3 | .4% | .9% |
| G40 Epilepsy and recurrent seizures | 3 | .4% | .9% |
| K52 Other and unspecified noninfective gastroenteritis and colitis | 3 | .4% | .9% |
| L89 Pressure ulcer | 3 | .4% | .9% |
| L03.90 Cellulitis, unspecified | 3 | .4% | .9% |
| K56.0 Paralytic ileus | 3 | .4% | .9% |
| G62.9 Polyneuropathy, unspecified | 3 | .4% | .9% |
| F03 Unspecified dementia | 2 | .3% | .6% |
| I27.20 Pulmonary hypertension, unspecified | 2 | .3% | .6% |
| B19.1 Unspecified viral hepatitis B | 1 | .1% | .3% |
| B45.1 Cerebral cryptococcosis | 1 | .1% | .3% |
| I71.9 Aortic aneurysm of unspecified site, without rupture | 1 | .1% | .3% |
| J44.9 Chronic obstructive pulmonary disease, unspecified | 1 | .1% | .3% |
| R04.0 Epistaxis | 1 | .1% | .3% |
| Q61.2 Polycystic kidney, adult type | 1 | .1% | .3% |
| Q44.6 Cystic disease of liver | 1 | .1% | .3% |
| Q39.4 Esophageal web | 1 | .1% | .3% |
| K75.0 Abscess of liver | 1 | .1% | .3% |
| K74.60 Unspecified cirrhosis of liver | 1 | .1% | .3% |
| I72.8 Aneurysm of other specified arteries | 1 | .1% | .3% |
| G43 Migraine | 1 | .1% | .3% |
| G30 Alzheimer's disease | 1 | .1% | .3% |
| B02 Zoster [herpes zoster] | 1 | .1% | .3% |
| I26 Pulmonary embolism | 1 | .1% | .3% |
| I82.40 Acute embolism and thrombosis of unspecified deep veins of lower extremity | 1 | .1% | .3% |
| M43.00 Spondylolysis, site unspecified | 1 | .1% | .3% |
| F20 Schizophrenia | 1 | .1% | .3% |
| B37.9 Candidiasis, unspecified | 1 | .1% | .3% |
| Total | 780 | 100.0% | 232.1% |
